# How to Eat

**Published:** 1905-06-10

**Authors:** 


### HOW TO EAT.

Certainly the human race seems to be in danger of the gradual extinction which is a corollary of over-specialisation. We have for years been hearing sermons on the great problem "What to eat," and now we are being provided with hints on the absorbing topic indicated by the heading of this article. The distressing feature in all these matters is that there is a good deal to be said for the new doctrines; the bulk of us are so civilised that no greater departure from a natural animal existence can be imagined than this which falls to the lot of workers in great centres of industry. Thus it happens that while the robust countryman may cry down all these new-fangled teachings as mere idle fancies, and point with assurance to himself and his lucky partners in outdoor living, whether beast or man, as evidence of their futility, the medical profession remains painfully aware that the analogy is false. So unnatural to the human animal is the indoor life of the stool and office that the victims of it become a law unto themselves, and such broad generalisations as that indicated above entirely beg the question.

Under the alluring title "Is man poltophagic or psomophagic?" Mr. Hubert Higgins[1] introduces us to two apparently distinct varieties of deglutition, πόλτος means "porridge," and ψωμός means "a morsel," hence a poltophagic animal is one that swallows its food in a pultaceous condition, while a psomophagic creature disposes of its meal in large fragments. The question at issue is therefore whether man in a general way of speaking masticates his food as much as he should. Some years ago Mr. Horace Fletcher and Mr. E. H. van Someren conducted an investigation into the subject upon which Sir Michael Foster reported as follows. "The adoption of the habit of thorough insalivation of food was found, in a consensus of opinion, to have an immediate and very striking effect upon appetite, making this more discriminating and leading to the choice of a more simple dietary, and, in particular, reducing the craving for flesh food. The appetite, too, is beyond all question fully satisfied with a dietary which had a total considerably less in amount than with ordinary habits is demanded." There is thus a prima facie case for consideration of the relative merits of the two methods of eating.

It appears that the pharyngeal anatomy of the poltophagic is different from that of the psomophagic varieties of animal. For instance, the horse, which may be regarded as a typical poltophagist, has a soft palate which is thick and muscular, and extends centrally well down in front of the large and rounded epiglottis. This arrangement is of course well adapted to the swallowing of the long, fusiform, semi-solid masses with which it is called upon to deal. How closely the soft palate is related to the tongue and epiglottis in the horse is shown by the inability of this animal to breathe through its mouth. The dog on the other hand is a type of the psomophagist. In this creature the soft palate is a thin translucent membrane almost devoid of muscle-fibres, which seems to serve no other purpose than that of a valve to close the posterior nares during the act of deglutition; the opening of the pharynx is large, and the epiglottis pointed and thin. "The well-known fact that the dog laps water shows that it lacks the power of suction by the soft palate."

Mr. Higgins holds that man is a poltophagist. By means of some ingenious experiments he has secured records of the voluntary movements of which the soft palate in man is to be considered capable. To illustrate the poltophagic method of mastication and deglutition he traces the course of events when a competent poltophagist devotes his attention to a piece of currant cake. After a small amount of mastication has taken place the following is the sequence of events: "As the starch is converted into dextrose it is dissolved by the saliva. From time to time samples of the fluid contents of the anterior buccal cavity are withdrawn (by the action of the soft palate and tongue) into the buccal passage, where it passes on to the posterior buccal cavity. When sufficient has collected a swallowing impulse is excited. . . . When the process of mastication and deglutition is completed there is nothing left but some almost dry currant skins and stones."

It is claimed that by the establishment of poltophagic habits of eating the appetite becomes well-defined at the commencement of a meal, and ceases definitely at the end; that is to say, there is no unholy hankering after nuts and wine or preserved fruits; there is marked increase in the pleasure of eating; the taste for simple food becomes pronounced, and preferences for particular chemical elements of the simple dietary are more defined; the fæces are lessened in amount and become odourless; the large intestine becomes smaller, and the fæces tend to remain longer in the bowel. "In all cases it can be said that there is an extraordinary change in the general health, much more joy of living, increased power of work, and freedom from the infinitely troublesome concomitants of chronic illness."

Reduced to its simplest terms poltophagy seems to be second cousin to vegetarianism, and will, no doubt, claim its adherents like every other innovation. Those who are well will probably laugh at it, while those who are dissatisfied with their state of health will equally probably try the new method and feel better. The truth would seem to be that the sedentary human being requires alteratives at intervals, whether they be medicinal, or dietetic, or administered in the form of a change of scene or of occupation; and if a man is unable to take a holiday at a time when he is afflicted with the minor, but very real, discomforts which lead him to say in the common phrase that he does not "feel fit," he may well do worse than attempt the new art of eating which has been described above.

[1] Lancet, May 20 and 27, 1905.

